# Rapid detection of porcine kobuvirus in feces by reverse transcription loop-mediated isothermal amplification

**DOI:** 10.1186/1743-422X-11-73

**Published:** 2014-04-23

**Authors:** Changlong Li, Jianfei Chen, Hongyan Shi, Xin Zhang, Da Shi, Xiao Han, Yanbin Chi, Li Feng

**Affiliations:** 1 Division of Swine Infectious Diseases, State Key Laboratory of Veterinary Biotechnology, Harbin Veterinary Research Institute of the Chinese Academy of Agricultural Sciences, No.427 Maduan Street, Nangang District, Harbin 150001, China; 2 College of Life Science, Northeast Agricultural University, Harbin 150030, China

**Keywords:** Porcine kobuvirus, Reverse transcription loop-mediated isothermal amplification (RT-LAMP), RT-PCR

## Abstract

**Background:**

PKV is a new emerging pathogen detected in diarrhea pigs. At present, no more detection methods were reported except RT-PCR method. this study was to develop a fast diagnostic method based on the LAMP reaction for rapid detection of PKV nucleic acid in fecal samples.

**Findings:**

Two pairs of primers were designed to amplify the conservative 3D gene of PKV genome. The PKV RT-LAMP method possessed well specificity and had 100 times higher sensitivity than common reverse transcription PCR (RT-PCR), which could detect up to 10 RNA copies of the target gene.

**Conclusions:**

The results showed that the optimal reaction condition for RT-LAMP was achieved at 64°C for 50 min. Furthermore, the RT-LAMP procedure does not demand special equipment and is time-saving.

## Introduction

Kobuvirus belongs to the family *Picornaviridae*, a small and non-enveloped virus with a single stranded, positive-sense genomic RNA. The genus Kobuvirus consists of three species, Aichivirus A (formerly Aichi virus), Aichivirus B (formerly Bovine kobuvirus) and Aichivirus C (aka porcine kobuvirus). Kobuviruses have a wide range of host species including humans [1], cattle [2], pigs [3], sheep [4], bats [5], canine [6], cats [7], ferrets [8] and goats [9].

PKV was first identified from stool specimens of clinically healthy and diarrhea domestic pigs in 2007 in Hungary [10]. In the past several years PKV has been identified in China [11], Thailand [12], Japan [13], Korea [14], USA [15],Italy [16] and Brazil [17].

During the past three years, China has suffered from severe porcine diarrhea and serious economic loss. Apart from the three main viruses, Porcine Epidemic Diarrhea Virus (PEDV), Transmissible Gastroenteritis Virus (TGEV) and A group Porcine Rotavirus (PoRV), which causing porcine diarrhea, PKV was identified from the diarrhea pigs, separately existed or mixed infected with the three viruses. Recent studies indicated that PKV was involved in swine diarrhea and may act as a special part in gastroenteritis pathogenesis [10]. Early detection and diagnosis can serve to control the spread of PKV and decrease economic loss.

Most studies investigating the distribution and prevalence of PKV employed reverse transcription-polymerase chain reaction (RT-PCR) method to amplify the conserved region, which targeting the 3D region of PKV genome [18,19]. At present, no more detection methods were reported except RT-PCR method. Sequence analysis indicated the 3D region is the most conservative, which suggests that the 3D region could be selected as the target sequence for loop-mediated isothermal amplification detection to investigate the distribution and prevalence of PKV.

As PCR requires an expensive thermal cycler and operator skill, which are limited to the field, a novel technique, loop-mediated isothermal amplification (LAMP) was developed by Notomi et al. [20] to overcome the difficulties of PCR-based techniques. RT-LAMP assays are more sensitive than conventional gel-based RT-PCR assays, fast and easy to perform since they require only a simple incubator, such as a heating block or a water bath to provide a constant temperature for the reaction [21]. This technique just requires only 30 to 60 minutes and can be performed at a single temperature ranging from 60°C to 65°C. Therefore, this study was to develop a fast diagnostic method based on the LAMP reaction for rapid detection of PKV nucleic acid in fecal samples.

## Materials and methods

### Primers for RT-LAMP and RT-PCR

LAMP primers were designed using Primer Explorer V4 software (Eiken Chemical Co., Ltd., Japan) (Table 1). The PKV (CH/HZ/2011, JX827598) specific primers used for RT-PCR were: sense PKV-S1 (5’-GGAAGAGGCGATCAATGGAAC-3’) (nucleotide location: 6805–6825) and antisense PKV-R1 (5’-GGCGTTCGAGGTGTTTCTCAAC-3’) (nucleotide location: 7277–7298) targeting a 494 bp fragment of the 3D region of PKV genome.

**Table 1:** LAMP primers for detection of PKV

**Primer name**	**Length**	**Type**	**Genome position**	**Sequence (5'-3')**
F3	18	Forward outer	7525-7342	CCCGCTACATCGAGACCA
B3	20	Reverse outer	7530-7549	TGGGTGGATCACACCCATAG
FIP	42	Forward inner	F1:7395-7416	GCCAACACATCCAGACGGGTTA-
			F2:7350-7369	CTCACGCCATGTCTTTGGTA
BIP	42	Reverse inner	B1:7450-7471	TGTGTCCTCTCCGCTCTGATCC-
			B2:7510-7529	ATCACATCATCACCGTAGGC

The position of LAMP primers are shown relative to the CH/HZ/2011 strain PKV (Accession No. JX827598).

### RNA extraction

135 Fecal samples (from Heilongjiang, Jilin, Jiangxi, Jiangsu and Guangdong province) were collected from Diagnostic Center of Harbin Veterinary Research Institute and stored at −20°C. Stool specimens were converted to 10% (w/v) suspensions in PBS (0.01 M phosphate, pH 7.2-7.4, 0.15 M NaCl) for RNA extraction. Total RNA was extracted from 400 μl suspensions using TRIZOL reagent (Invitrogen, Carlsbad, CA, USA) according to the manufacturer's instructions. The RNA was stored at −80°C until use.

### RT-LAMP and RT-PCR assays

The RT-LAMP was carried out in a volume of 25 μl. The optimized reaction mixture contains 40 pmol of inner primers FIP and BIP, 5 pM of outer primers F3 and B3, 35 pM dNTP (TaKaRa, Japan), 1× ThermoPol buffer (New England Biolabs, UK), 6 mM MgSO4 (TaKaRa, Japan), 1 μl betaine (Sigma, USA) and 8 U of Bst DNA polymerase (large fragment; New England Biolabs), 20 U M-MLV reverse transcriptase (TaKaRa, Japan) and 1 μl of template.

To determine the optimal reaction temperature, the RT-LAMP reaction mixtures were incubated at 62, 63, 64 and 65°C for 60 min. The optimal reaction time was determined by performing the RT-LAMP at the optimal temperature for 30, 40, 50 and 60 min. Finally, the reaction was terminated by heat inactivation at 80°C for 5 min.

The RT-PCR amplification was carried out in a 50 μl reaction mix by One-step RNA PCR Mix (AMV) (TaKaRa, Japan) according to the manufacturer's instructions. The RT-PCR was as follows: 50°C 30 min; 94°C 2 min; 94°C 30 s, 60°C 30 s, 72°C 30 s, 30 cycles; 72°C 6 min. RT-LAMP and RT-PCR products were electrophoresed through a 2% agarose gel after reaction.

### Sensitivity between RT-LAMP and RT-PCR

To compare the sensibilities of RT-PCR and RT-LAMP, F3/B3 and PKV-S1/R1 primers were used to amplify the target fragments and linked to pET-30a vector (with T7 promoter), then sequenced as the templates for RNA transcription. The standard RNAs were transcribed from the plasmid DNAs by using mMESSAGE mMACHINE T7 kit (Ambion). The RNAs were calculated the RNA copy numbers and diluted with different gradients (10^9^ ~ 10° copies/μl). The RNAs with gradient dilution were regarded as the templates for the comparison of sensitivity between RT-PCR and RT-LAMP detection.

### Specificity of RT-LAMP and RT-PCR

For confirmation of the specificity of the RT-LAMP products, PEDV, TGEV, A group PoRV, which could cause pig diarrhea, two members of porcine picornaviruses PEV 9 (Porcine Enterovirus 9) and PTV (Porcine Teschovirus) were also added to the specificity assay. The above mentioned RNA templates were tested by RT-LAMP reactions.

## Results

### RT-LAMP detection of PKV

**Figure 1:**
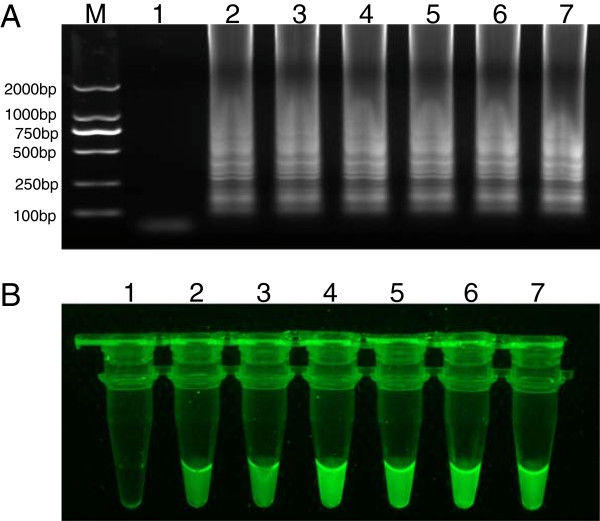
**Detection of fecal samples from different pig farms by RT-LAMP method. A**: Electrophoretic analysis of RT-LAMP products. M, 2000 bp DNA marker; Lane 1, negative control; Lane 2–7, fecal samples from different pig farms. **B:** Visual inspection of the RT-LAMP. Tube 1, negative control; Tube 2–7, fecal samples from different pig farms.

RT-LAMP products were electrophoresed through a 2% agarose gel, which yielded typical ladder-like bands (Figure 1A). For visual detection of LAMP products, SYBR green I dye (diluted 1:1000) was added to LAMP reaction and exposed to UV light. The positive LAMP showed green fluorescence while the negative control was with no green fluorescence (Figure 1B).

### Optimal temperature and time for the PKV RT-LAMP assay

**Figure 2:**
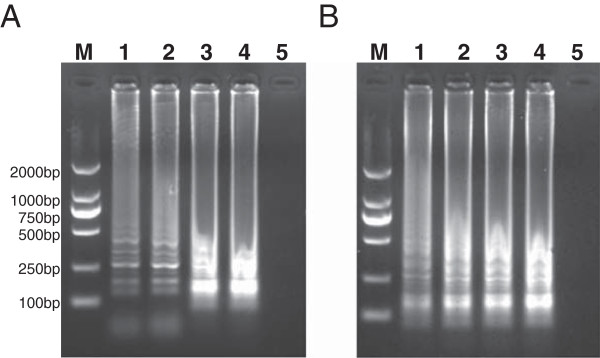
**Optimal temperature and time for the PKV RT-LAMP assay.** The optimization of the PKV RT-LAMP was analyzed by performing the reaction at 62, 63, 64 or 65°C for 1 h. The reaction results are shown in lanes 1–4, respectively **(A)**. The same reaction was performed at 64°C for 30, 40, 50, or 60 min. The RT-LAMP results are shown in lanes 1–4, respectively **(B)**. Lane M: DNA marker; lane 5: Negative control.

The optimal reaction temperature and incubation time of the RT-LAMP were investigated. The DNA products of the RT-LAMP at different temperatures showed multiple of characteristic ladder bands. The agarose gel analysis indicated that the intensity of DNAs at 64°C was stronger than other reaction temperatures (Figure 2A). Then RT-LAMP was performed at 64°C for different time points. As shown in Figure 2B, the DNA product showed the highest intensity when the reaction was performed for 50 min. Therefore, the optimal reaction condition of the PKV RT-LAMP was 64°C for 50 min.

### Sensitivity between RT-LAMP and RT-PCR

**Figure 3:**
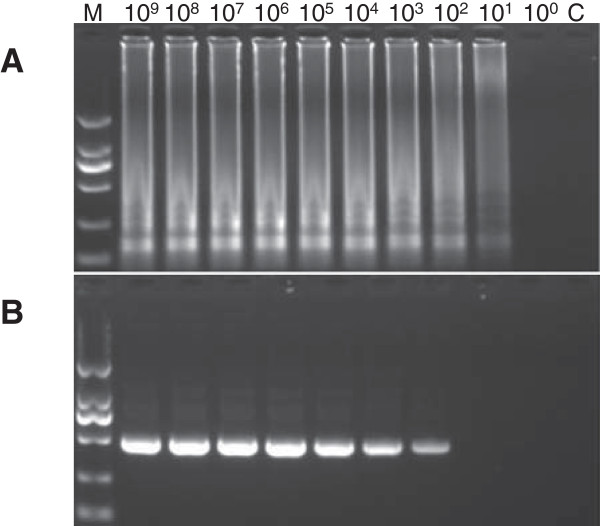
**Comparison of sentivities of LAMP and RT-PCR methods for the detection of PKV by 2% agarose gel electrophoresis.** The RNAs was serially diluted and calculated copy numbers. Each RNA concentration was subjected to the RT-LAMP and RT-PCR assays. **(A)** RT-LAMP assay. **(B)** RT-PCR amplification. M, 2000 bp DNA marker.

The clones contains the target fragments were diluted from 10^9^ ~ 10° copies/μl. The detection limit of RT-LAMP was 10^1^ copies (Figure 3A) while RT-PCR was 10^3^ copies (Figure 3B).

### Specificity of RT-LAMP and RT-PCR

**Figure 4:**
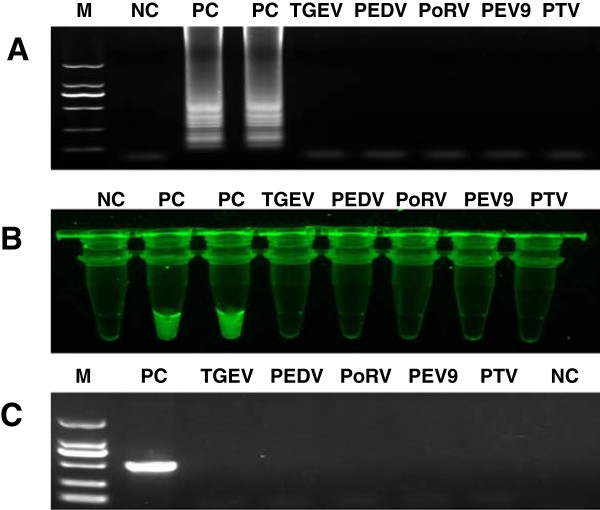
**The specificity of RT-LAMP and RT-PCR. (A)** The RNAs of TGEV, PEDV, A group PoRV, PEV 9 and PTV were added to the RT-LAMP reaction, no ladder-like bands appeared. M, 2000 bp DNA marker; **(B)** SYBR green I dye (1:1000, 1 μl) was added to LAMP reaction and exposed to UV light. Only the PKV tube showed green fluorescence. **(C)** The specificity of RT-PCR. PC: positive control; NC, negative control.

The specificity of RT-LAMP was showed in Figure 4, which demonstrated that the developed RT-LAMP and RT-PCR could specifically distinguish PKV genome, without cross-reaction to PEDV, TGEV, A group PoRV, PEV 9 and PTV (Figure 4).

### Concordance of RT-LAMP and RT-PCR

PKV RT-LAMP assay was compared to RT-PCR detection by utilizing 135 clinical feces samples. Results indicated that LAMP was more sensitive than RT-PCR method. The concordance of the two methods was 88.1% (Table 2).

**Table 2:** Comparison of concordance between LAMP and RT-PCR for detection of PKV

**Detection method**	**Positive**	**Negative**	**Total**	**Concordance**
LAMP	101	34	135	88.1%
RT-PCR	89	46	135	

## Discussion

Porcine kobuvirus, a new species of the genus *Kobuvirus* in the family *Picornaviridae*, had been reported prevalent in many countries. The information about this virus is still limited and it is uncertain whether porcine kobuvirus is involved in pig diarrhea as it can be detected in both diarrhea and healthy pigs. Up to now, porcine kobuvirus has not been successful purified, which limited further study of the virus. More researches need to be done to reveal its feature and pathogenicity.

RT-PCR detection of the 3D region has been widely used for the identification and epidemiological study of porcine kobuvirus [14-16]. At present, RT-PCR is the unique detection method to detect the nucleic acid of PKV and no more detection methods were reported. Furthermore, RT-LAMP is used increasingly for clinical diagnosis of many pathogens including Newcastle disease virus, Salmonella enterica, porcine circovirus, and porcine parvovirus [21-24].

To summarize, the PKV RT-LAMP was established with high sensitivity and specificity. Compared to the RT-PCR method, this assay is with higher analytical and clinical sensitivity. As the RT-LAMP method is easy to set up and does not need additional equipment, it gets obvious advantages in clinical diagnosis better than the conventional PCR method.

## Competing interests

The authors declare that they have no competing interests.

## Authors’ contributions

CL and LF designed the experiment. CL carried out most of the experiments and wrote the manuscript. CL, XH and YC prepared the fecal samples and organized the data. DS participated in some of the experiments. JC, XZ and HS revised the manuscript. All the authors read and approved the final manuscript.
